# State-Level Tax Policy, Cancer Screening, and Mortality Rates in the US

**DOI:** 10.1001/jamanetworkopen.2025.8455

**Published:** 2025-05-02

**Authors:** Odysseas P. Chatzipanagiotou, Mujtaba Khalil, Usama Waqar, Selamawit Woldesenbet, Giovanni Catalano, Timothy M. Pawlik

**Affiliations:** 1 Department of Surgery, The Ohio State University Wexner Medical Center, Columbus; 2 Department of Surgery, Emory University, Atlanta, Georgia; 3 Department of Surgery, University of Verona, Verona, Italy

## Abstract

**Importance:**

The Healthy People 2030 initiative has set national cancer screening targets for breast, colon, and cervical cancers, as well as aims for reducing cancer mortality. State-level tax policy is an underappreciated social determinant of health that may improve cancer screening and mortality rates.

**Objective:**

To define the association of tax revenue and tax progressivity with state-level cancer screening and mortality.

**Design, Setting, and Participants:**

This ecologic, population-based, cross-sectional study assessed cancer screening (2020 and 2022) and mortality rates (1999-2021) in the US relative to state-level tax revenue (1997-2019) and tax progressivity (2002, 2009, 2012, 2014, and 2018) with a 2-year lag. The study included 50 states through 23 years with state-years used as the unit of analysis. Cancer screening rates were derived from the Centers for Disease Control and Prevention (CDC) Population Level Analysis and Community Estimates database. State-level cancer-related death and population counts were derived from the CDC Wide-Ranging Online Data for Epidemiologic Research database. Data analysis occurred from September to January 2024.

**Exposure:**

State-level tax policy was proxied by tax revenue per capita and the Suits index of tax progressivity, with progressive taxation equaling lower tax burden for more disadvantaged populations.

**Main Outcomes and Measures:**

Outcomes included screening rates for colon, breast, and cervical cancer, as well as mortality rates for all malignant neoplasms and malignant neoplasms with guideline-recommended screening. Multivariable models were adjusted for tax-related, socioeconomic, and demographic variables.

**Results:**

In total, 1150 state-years were included in the analysis. Median (IQR) tax revenue per capita was $4432 ($3862-$5210), and the median (IQR) number of cancer-related deaths was 8341 (3150-13 585) across all state-years. Of note, each $1000 increase in tax revenue per capita was associated with a 1.61% (95% CI, 0.50%-2.73%) increase in colorectal cancer screening, 2.17% (95% CI, 1.39%-2.96%) increase in breast cancer screening, and 0.72% (95% CI, 0.34%-1.10%) increase in cervical cancer screening rate. For malignant neoplasms with guideline-recommended screening, each $1000 increase in tax revenue per capita was associated with a decreased cancer mortality rate among White (adjusted incidence rate ratio, 0.95, 95% CI, 0.93-0.98), but not racial and ethnic minority (adjusted incidence rate ratio, 0.99, 95% CI, 0.97-1.02) populations.

**Conclusions and Relevance:**

In this cross-sectional study, tax policy was associated with increased state-level cancer screening rates, as well as decreased cancer mortality rates, which mostly benefited White populations, suggesting that state-level policies may contribute to bridging ongoing cancer care gaps.

## Key Points

**Question:**

Are state-level tax revenue and tax progressivity associated with cancer screening and cancer mortality rates in the US?

**Findings:**

This cross-sectional study of 1150 state-years consisting of tax revenue data for 23 years (1997-2019) and 50 states found that increased tax income was associated with increased cancer screening rates, as well as decreased cancer mortality rates; this association was more prominent among White than racial and ethnic minority populations.

**Meaning:**

These findings suggest that state-level tax revenue may serve as one aspect of a multifaceted approach to improve cancer-related outcomes in the US and help bridge cancer care gaps, particularly in more progressive tax policy settings.

## Teaser

This cross-sectional study assesses associations of state-level tax revenue and tax progressivity with state-level cancer screening and mortality in the US.

## Introduction

The Healthy People 2030 initiative set goals of implementing cancer screening and prevention programs, as well as elevating the level of care and improving survivorship among patients with cancer.^1,2^ Target population goals were set at 68.3% for colorectal cancer screening, 80.3% for breast cancer screening, and 79.2% for cervical cancer screening.^3,4,5^ Regarding overall cancer mortality, the aim is to reduce the baseline 149.1 cancer deaths per 100 000 population by approximately 30 deaths per 100 000 until the year 2030.^1^ Even though the number of cancer-related deaths has been declining in recent years, cancer persists as the second leading cause of death in the US, resulting in more than 600 000 deaths every year.^2,6,7^ Importantly, disparities persist despite screening initiatives, earlier diagnoses, and improved treatment options, especially among disadvantaged populations in underserved areas that consistently experience higher cancer incidence, lower screening rates, decreased odds of receiving guideline-appropriate treatment, and higher cancer death rates.^1,8^

Various social determinants of health (SDoH), including socioeconomic privilege, racial segregation, redlining, and food insecurity impact cancer screening and mortality.^9,10,11,12,13,14^ In theory, state-level tax policy can be considered an upstream SDoH because the generation of tax revenue and the redistribution of wealth through progressive tax policies may help mitigate socioeconomic and health care disparities.^15^ Of note, progressive taxes are designed to burden wealthier individuals more than less wealthy populations, which is opposite to regressive taxation.^15,16,17^ Previous studies assessing the impact of tax policy on health outcomes have focused on how the earned income tax credit and sin taxes on tobacco or alcohol have promoted health-related outcomes.^18,19^ However, few studies have considered the entire tax system as an SDoH. Specifically, a recent study by Junior et al^15^ assessed the association of state-level tax policy with infant mortality rates across the US, demonstrating the impact that tax policy had on this important indicator of population health.

According to the National Cancer Institute, screening and mortality rates are measures that reflect cancer health disparities.^8^ Nonetheless, the impact of state-level tax policy on cancer screening and mortality rates remains ill-defined. Therefore, the objective of the current study was to assess how state-level tax policy, including tax revenue and tax progressivity may be associated with cancer screening and mortality rates. We hypothesized that increased tax revenue and more progressive tax policies would be associated with higher screening rates and lower mortality rates.

## Methods

### Study Design and Data Sources

This population-based, state-level, cross-sectional study was deemed exempt from institutional review board approval and the requirement of informed consent by The Ohio State University because data were derived from publicly available data sources and analyzed at the state level without individual information. The study followed the Strengthening the Reporting of Observational Studies in Epidemiology (STROBE) reporting guideline.^20^ We utilized data from the US Census Bureau Annual Survey of State and Local Government Finances and the Institute on Taxation and Economic Policy (ITEP) to calculate state-level tax revenue and tax progressivity.^15,16,17,21,22,23,24,25^ The unit of analysis was the state-year, which represented the combination of a specific year and state for each outcome of interest (eFigure 1 in Supplement 1). Covariates included in the analysis were queried from the US Census Bureau and the Bureau of Economic Analysis.^26,27^ Tax policy data were made available upon request by Junior et al.^15^ Cancer mortality data from 1999 to 2021 were derived from the Centers for Disease Control and Prevention (CDC) Wide-Ranging Online Data for Epidemiologic Research (WONDER) database.^28^ Moreover, cancer screening rates for the years 2020 and 2022 were derived from the CDC Population Level Analysis and Community Estimates (PLACES) database, which includes model-based predictions for 29 health-related outcomes and behaviors at various geographic levels in the US (eMethods in Supplement 1).^9,29^ In the current study, county-level data were aggregated at the state level.

### Primary Exposure and Outcomes of Interest

The primary exposure was state-level tax policy, proxied by tax revenue per capita and the Suits index of tax progressivity, with higher progressivity representing increased taxation among wealthier individuals.^15,17^ Tax revenue per state was calculated as the sum of all revenue at the state and local level defined as taxes by the US Census Bureau.^30^ Local-level revenue collected by counties, cities, townships, special districts, and independent school districts or educational service agencies was aggregated at the state level. Five editions of tax progressivity data had been published by the ITEP within the study period, namely for 2002, 2009, 2012, 2014, and 2018, evaluating policies on income, property, and general sales taxes.^15,16,21,22,23,24,25^ The Suits index for tax progressivity was calculated based on previously established methods at the state level using ITEP data.^31,32^ The Suits index ranges from −1, corresponding to the most regressive tax policy, to 1, for the most progressive tax policy, and has been previously used to measure tax progressivity. For ease of interpretation, the Suits index was multiplied by a factor of 10 when reported in the current study, thus ranging between −10 and 10.^15,33,34^

The primary outcomes of interest included: (1) age-adjusted (2020 standard US population), state-level breast, colorectal, and cervical cancer screening rates; (2) state-level cancer mortality rates; and (3) state-level cancer mortality rates among cancers with guideline-recommended screening (ie, breast, cervical, and colorectal malignant neoplasms). Secondary outcomes included state-level cancer mortality for (1) White and (2) racial or ethnic minority only (ie, American Indian or Alaska Native, Asian or Pacific Islander, and Black or African American) populations.

### Covariates

The covariates included in the model were selected based on the methodology used previously when evaluating the association of tax policy as an SDoH with public health indicators. Covariates included federal transfer revenue per capita, other revenue per capita, health spending per capita, gross domestic product per capita, percentage of Hispanic population, percentage of non-Hispanic Black population, percentage of population aged 25 years or older with a high-school degree, as well as an interaction term between Medicaid expansion (ME) status (ME vs non-ME states) and a binary time variable related to the pre-ME and post-ME time periods that approximated a difference-in-difference term (eMethods in Supplement 1).^15,35,36^ The US Department of Commerce price indexes were used to adjust for inflation all government revenue and median household income data (2020 US dollars).^27^

### Statistical Analysis

The intraclass correlation and variance partition coefficient was 0.73 for colorectal cancer screening, 0.83 for breast cancer screening, 0.59 for cervical cancer screening, and 0.91 for cancer mortality outcomes, indicating that most of the variance was due to between-state rather than within-state differences. Generalized estimating equation (GEE) models were employed to account for clustering.^37^ The specific distribution and correlation structure were chosen based on the outcomes’ distributions and lower quasi-likelihood under the independence model criterion values among potential models (eMethods, eTable 1, and eFigure 2 in Supplement 1). Univariable and multivariable models were used to assess the association of the tax policy components—tax revenue and tax progressivity—and state-level cancer screening and cancer mortality. Results were reported as percentage estimates and incidence rate ratios (IRRs) with 95% CIs. The main analysis incorporated a 2-year lag representing the time required for the implementation and observed effects of tax policies (eMethods in Supplement 1).^15,38^ Multivariable models were adjusted for relevant economic and demographic variables, as well as a dummy variable for the year corresponding to the reported outcome. A Pearson correlation matrix was used to assess the associations among variables with a variance inflation factor threshold set at 5 (eFigure 3 in Supplement 1). A subanalysis was conducted using only state-years with available ITEP tax progressivity data, and sensitivity analyses were performed to assess different lag times, as well as state fixed effects (eTable 2 and eTable 3 in Supplement 1). Secondary analyses employed similar GEE models for cancer mortality, focusing on race- and ethnicity-specific cancer mortality rates. All tests were 2-tailed, and a *P* value of .05 was deemed significant. All statistical analyses were performed using R version 4.4.1 (R Foundation for Statistical Computing) and Python version 3.12.5 (Python Software Foundation)^39^ from September to January 2024.

## Results

### Descriptive Statistics

Overall, 1150 state-years consisting of tax revenue data for 23 years (1997-2019) and 50 states were included in the analysis (Figure, A-B). Among screening rates (100 state-years) for the years 2020 and 2022, the highest and mean (SD) prevalence of colon cancer screening was reported in Rhode Island (76.6% [4.7%]), while the lowest was reported in Alaska (60.3% [6.5%]). The highest mean (SD) prevalence for breast cancer screening was reported in Rhode Island (79.1% [2.4%]), with the lowest being in Alaska (63.1% [2.9%]); cervical cancer screening was most prevalent in Rhode Island (mean [SD] prevalence, 86.4% [1.0%]) and least prevalent in Arizona (mean [SD] prevalence, 79.0% [2.8%]). Of note, across the US in 2020, the median (IQR) prevalence was 64.8% (62.2%-67.2%) for colon cancer screening, 71.6% (68.8%-74.5%) for breast cancer screening, and 83.5% (82.5%-84.5%) for cervical cancer screening, while in 2022, the median (IQR) prevalence was 71.5% (68.8%-74.6%) for colon cancer screening, 71.2% (67.9%-73.7%) for breast cancer screening, and 81.4% (79.9%-82.5%) for cervical cancer screening (Figure, C).

**Figure.**
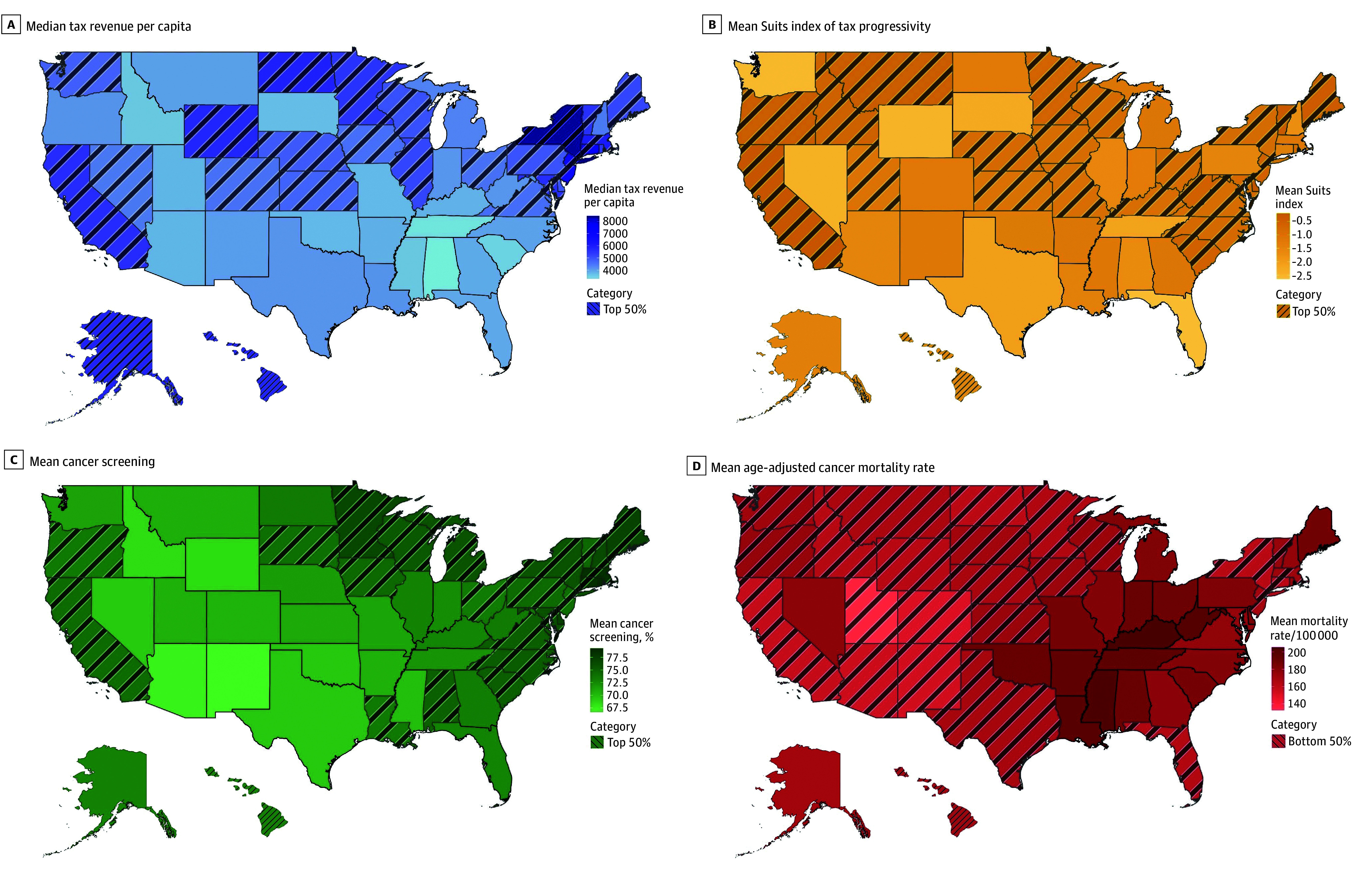
Choropleth Maps of the US Illustrating State-Level Variability The figure shows variability in median tax revenue per capita (1997-2019; A), mean Suits index of tax progressivity (2002, 2009, 2012, 2014, and 2018) multiplied by a factor of 10 for ease of interpretation (B), mean cancer screening percentage (2021) for breast, cervical, and colorectal cancer (C), and mean age-adjusted cancer mortality rate (1999-2021; D). States in the top 50% for median tax revenue per capita, mean Suits index of tax progressivity, and mean cancer screening percentage, as well as those in the bottom 50% for mean age-adjusted cancer mortality rate, are indicated with diagonal lines.

Across all 1150 state-years with tax revenue data, there was a total of 13 223 914 cancer-related deaths (median [IQR], 8341 [3150-13 585] cancer-related deaths) and an overall age-adjusted cancer mortality rate of 173.6 deaths per 100 000 population (Figure, D). The highest and lowest age-adjusted cancer mortality rates were 230.6 and 119.4 deaths per 100 000 population in Louisiana (1999) and Utah (2021), respectively. Kentucky was the state with the highest mean (SD) age-adjusted mortality rate, with 205.2 (16.2) deaths per 100 000 population, while Utah demonstrated the lowest mean (SD) age-adjusted mortality rate, with 132.7 (10.7) deaths per 100 000 population (Table 1). By race and ethnicity, the mean (SD) age-adjusted cancer mortality was 205.7 (39.8) per 100 000 non-Hispanic Black population, 174.9 (20.3) per 100 000 non-Hispanic White population, and 107.2 (32.2) per 100 000 Hispanic population.

**Table 1.** State Tax Revenue Per Capita (1997-2019), Suits Index of Tax Progressivity (2002, 2009, 2012, 2014, and 2018), Screening Rate (2020 and 2022), Death Count, and Mortality Rate (1999-2021)

State	Tax revenue per capita, median (IQR), $ (thousands)^a^	Suits index, mean (SD)^b^	Colorectal, breast, and cervical screening rate, median (IQR), %	Death count, mean (SD)	Age-adjusted cancer mortality rate, mean (SD) per 100 000 population
Alabama	3.3 (3.0-3.5)	−1.62 (0.24)	74.1 (71.8-78.7)	10 129 (297)	188.9 (18.2)
Alaska	5.2 (4.7-9.4)	−1.41 (0.42)	63.1 (61.7-71.1)	873 (132)	174.1 (18.1)
Arizona	3.8 (3.7-3.9)	−1.48 (0.22)	63.8 (62.4-71.4)	10 742 (1230)	154.3 (14.5)
Arkansas	3.9 (3.3-4.0)	−1.12 (0.21)	68.9 (67.0-74.8)	6416 (188)	192.7 (15.2)
California	5.5 (4.9-6.0)	−0.04 (0.46)	70.5 (67.7-76.2)	56 564 (2499)	156.5 (16.8)
Colorado	4.5 (4.3-4.7)	−1.15 (0.30)	67.1 (66.1-74.9)	7034 (737)	149.4 (15.5)
Connecticut	7.1 (6.4-7.8)	−1.26 (0.34)	77.2 (76.2-81.7)	6829 (231)	161.3 (19.2)
Delaware	4.9 (4.7-5.1)	−0.17 (0.31)	76.3 (73.8-80.0)	1904 (163)	182.7 (21.0)
Florida	3.8 (3.7-4.1)	−2.69 (0.24)	71.3 (70.0-76.0)	42 012 (2549)	164.0 (16.7)
Georgia	3.9 (3.7-4.0)	−1.08 (0.26)	73.1 (69.4-77.5)	15 616 (1531)	176.8 (18.3)
Hawaii	5.8 (4.8-6.2)	−1.06 (0.21)	76.3 (73.4-79.2)	2261 (195)	139.7 (12.2)
Idaho	3.5 (3.4-3.8)	−0.50 (0.15)	64.7 (64.3-72.0)	2572 (332)	161.0 (13.6)
Illinois	5.1 (4.6-6.0)	−1.49 (0.31)	71.5 (69.3-76.4)	24 342 (401)	179.2 (19.4)
Indiana	4.1 (3.8-4.2)	−1.25 (0.14)	69.0 (67.8-75.6)	13 180 (354)	188.7 (16.6)
Iowa	4.5 (4.0-4.9)	−0.84 (0.13)	73.4 (71.8-78.2)	6406 (77)	171.5 (13.4)
Kansas	4.7 (4.2-4.9)	−1.03 (0.36)	69.0 (66.6-75.5)	5427 (110)	172.2 (12.9)
Kentucky	3.8 (3.6-3.9)	−0.91 (0.11)	69.1 (68.8-75.1)	9768 (421)	205.2 (16.6)
Louisiana	4.1 (3.7-4.5)	−1.34 (0.21)	74.5 (70.6-78.5)	9298 (204)	196.9 (21.7)
Maine	5.2 (4.9-5.3)	−0.38 (0.22)	74.4 (74.3-79.6)	3214 (124)	185.6 (18.5)
Maryland	5.6 (5.1-6.0)	−0.68 (0.29)	74.2 (72.5-79.4)	10 477 (244)	173.6 (22.5)
Massachusetts	6.0 (5.4-6.4)	−1.14 (0.25)	79.0 (77.3-82.1)	13 088 (470)	170.5 (23.2)
Michigan	4.3 (4.2-4.5)	−1.00 (0.26)	73.4 (72.7-78.5)	20 395 (511)	180.3 (15.2)
Minnesota	5.4 (5.1-6.2)	−0.53 (0.36)	74.8 (73.6-79.0)	9487 (397)	163.7 (15.8)
Mississippi	3.5 (3.2-3.8)	−1.17 (0.15)	68.3 (66.7-75.8)	6294 (240)	200.7 (14.3)
Missouri	3.8 (3.7-3.9)	−0.89 (0.15)	68.5 (67.4-74.7)	12 642 (307)	185.2 (15.4)
Montana	3.9 (3.4-4.2)	−0.42 (0.17)	68.0 (66.3-75.0)	1983 (102)	166.1 (18.3)
Nebraska	4.9 (4.4-5.2)	−0.79 (0.24)	68.3 (67.2-75.0)	3437 (74)	167.0 (13.0)
Nevada	4.4 (4.2-4.7)	−2.39 (0.30)	65.2 (64.4-73.3)	4585 (573)	176.4 (20.5)
New Hampshire	4.4 (4.0-4.8)	−1.73 (0.20)	72.9 (72.7-78.8)	2630 (141)	174.1 (20.1)
New Jersey	6.8 (5.7-7.2)	−0.46 (0.35)	74.1 (70.7-78.9)	16 820 (814)	168.1 (25.1)
New Mexico	4.0 (3.7-4.2)	−1.16 (0.22)	63.2 (62.1-71.5)	3330 (278)	152.6 (13.5)
New York	8.4 (6.6-9.1)	−0.74 (0.35)	75.2 (73.3-79.9)	35 420 (1202)	160.6 (21.0)
North Carolina	4.0 (3.8-4.2)	−0.76 (0.15)	74.1 (72.3-79.1)	17 992 (1500)	178.9 (17.1)
North Dakota	5.7 (3.9-7.8)	−1.36 (0.26)	73.1 (69.7-77.7)	1307 (42)	159.9 (16.4)
Ohio	4.5 (4.4-4.8)	−0.91 (0.14)	71.8 (69.5-77.2)	25 130 (224)	187.3 (16.8)
Oklahoma	3.6 (3.5-3.9)	−1.28 (0.17)	67.3 (63.9-73.6)	7829 (391)	190.9 (10.4)
Oregon	4.1 (3.8-4.4)	−0.33 (0.21)	70.8 (70.5-77.0)	7649 (461)	173.3 (16.7)
Pennsylvania	4.9 (4.3-5.1)	−1.42 (0.20)	72.0 (71.9-77.2)	28 963 (750)	179.6 (18.3)
Rhode Island	5.4 (4.8-5.6)	−0.78 (0.28)	79.1 (77.8-82.7)	2258 (108)	173.6 (20.8)
South Carolina	3.5 (3.3-3.6)	−0.59 (0.17)	75.1 (72.9-79.0)	9378 (872)	183.9 (18.1)
South Dakota	3.7 (3.4-3.9)	−2.34 (0.13)	73.5 (70.7-78.4)	1633 (64)	169.2 (13.9)
Tennessee	3.4 (3.2-3.5)	−2.16 (0.08)	70.2 (69.0-76.0)	13 458 (810)	193.4 (17.1)
Texas	4.0 (3.8-4.3)	−2.09 (0.12)	68.8 (65.2-73.9)	37 114 (3143)	167.3 (17.8)
Utah	3.8 (3.6-4.1)	−0.95 (0.35)	68.2 (67.4-73.9)	2800 (377)	132.7 (10.5)
Vermont	5.5 (4.5-5.9)	−0.30 (0.30)	72.4 (71.1-78.0)	1315 (80)	172.8 (15.9)
Virginia	4.6 (4.3-4.9)	−0.83 (0.19)	74.6 (72.1-79.3)	14 312 (711)	174.8 (20.3)
Washington	4.8 (4.5-5.1)	−2.55 (0.13)	68.8 (68.0-74.8)	11 837 (844)	169.6 (18.0)
West Virginia	4.1 (3.6-4.2)	−0.54 (0.13)	71.6 (69.9-76.9)	4704 (78)	198.5 (14.7)
Wisconsin	5.0 (4.8-5.1)	−0.74 (0.18)	72.6 (69.6-77.8)	11 154 (324)	171.2 (15.1)
Wyoming	5.8 (5.0-7.0)	−2.54 (0.38)	63.9 (62.4-72.8)	945 (65)	162.1 (18.4)

^a^ 2020 inflation adjusted.

^b^ Multiplied by a factor of 10 for ease of interpretation.

Over 23 years and across 50 states, the median (IQR) tax revenue per capita was $4432 ($3862-$5210), with the highest tax revenue per capita reported in Alaska in 2008 ($17 240), and the lowest in Alabama in 1998 ($2796). Specifically for the 250 state-years with available tax progressivity data, the median (IQR) tax revenue per capita was $4449 ($3891-$5351) and the mean (SD) Suits index was −1.12 (0.68). Interestingly, the vast majority of state-years (244 state-years [97.6%]) had regressive taxes, while the 5-year mean Suits index was negative in all US states (Table 1). Of note, only California in 2012, 2014, and 2018 as well as Delaware, Minnesota, and Vermont in 2018 had progressive tax policies according to the Suits index of tax progressivity.

### Tax Policy and Cancer Screening

Regarding the association of tax policies with screening rates with a 2-year lag, univariable analysis found that each $1000 increase in tax revenue per state resident was associated with an increase in prevalence of cancer screening by 1.95% (95% CI, 0.96%-2.95%) for colorectal cancer, 1.76% (95% CI, 0.98%-2.53%) for breast cancer, and 0.68% (95% CI, 0.35%-1.01%) for cervical cancer. Furthermore, each 0.10 increase in the Suits index was associated with a 2.73% (95% CI, 0.63-4.87%) increase in prevalence of colorectal cancer screening, a 2.48% (95% CI, 0.65%-4.35%) increase in prevalence of breast cancer screening, and a 1.04% (95% CI, 0.42%-1.67%) increase in prevalence of cervical cancer screening. Multivariable analysis, after adjusting for relevant factors, found that each $1000 increase in tax revenue per capita was associated with a 1.61% (95%CI, 0.50%-2.73%) increase in prevalence of colorectal cancer screening, a 2.17% (95% CI, 1.39%-2.96%) increase in prevalence of breast cancer screening, and a 0.72% (95% CI, 0.34%-1.10%) increase in prevalence of cervical cancer screening (Table 2).

**Table 2.** Univariable and Multivariable Generalized Estimating Equations Models for Cancer Screening Rates (2020 and 2022) With 2-Year Lag (100 State-Years)

Model	Change in colorectal cancer screening, % (95% CI)	*P* value	Change in breast cancer screening, % (95% CI)	*P* value	Change in cervical cancer screening, % (95% CI)	*P* value
Univariable						
Tax revenue per capita^a^	1.95 (0.96 to 2.95)	<.001	1.76 (0.98 to 2.53)	<.001	0.68 (0.35 to 1.01)	<.001
Tax progressivity^b^	2.73 (0.63 to 4.87)	.01	2.48 (0.65 to 4.35)	.008	1.04 (0.42 to 1.67)	.001
Multivariable^c^						
Tax revenue per capita^a^	1.61 (0.50 to 2.73)	.004	2.17 (1.39 to 2.96)	<.001	0.72 (0.34 to 1.10)	<.001
Tax progressivity^b^	0.61 (−1.80 to 3.08)	.62	1.46 (−0.15 to 3.08)	.08	0.56 (−0.16 to 1.28)	.13

^a^ For each $1000 increase in tax revenue per capita.

^b^ For each 0.10 unit increase in the Suits tax progressivity index.

^c^ Adjusted for federal transfer revenue per capita, other revenue per capita, non-Hispanic Black population percentage, high school graduation rate, health spending per capita, and Medicaid expansion (variance inflation factor <5).

### Tax Policy and Cancer Mortality

Among the entire 1150 state-years, univariable GEE analysis demonstrated an association of tax revenue per capita with cancer mortality (IRR, 0.96; 95% CI, 0.95-0.98). After adjusting for relevant tax-related and demographic variables, each $1000 increase in tax revenue was associated with a 2% decrease in cancer mortality rate (adjusted IRR [aIRR], 0.98; 95% CI, 0.95-0.98), as well as a 3% decrease in cancer mortality rate for White individuals (aIRR, 0.97; 95% CI, 0.95-0.98) (Table 3). Specifically, among years with available tax progressivity data, each $1000 increase in tax revenue remained associated with a 3% decrease in mortality rate among all cancers (aIRR, 0.97; 95% CI, 0.96-0.99), in addition to a 3% decrease in cancer mortality rate among White populations (aIRR, 0.97; 95% CI, 0.96-0.99), while there was no association with cancer mortality among minority populations (eTable 4 in Supplement 1).

**Table 3.** Univariable and Multivariable Generalized Estimating Equations Models for Cancer Mortality Rates (1999-2021) With 2-Year Lag (1150 State-Years) Using Poisson Distribution (Log Link) With Robust Standard Errors and an Exchangeable Correlation Matrix^a^

Model	Cancer mortality					
	All, aIRR (95% CI)	*P* value	White, aIRR (95% CI)	*P* value	Racial and ethnic minority, aIRR (95% CI)^b^	*P* value
Univariable						
Tax revenue per capita^c^	0.96 (0.95-0.98)	<.001	0.97 (0.96-0.98)	<.001	1.00 (0.97-1.02)	.77
Federal transfer revenue per capita^c^	0.96 (0.93-0.98)	.001	0.97 (0.95-0.99)	.005	0.98 (0.95-1.02)	.37
Other revenue per capita^c^	1.00 (0.99-1.00)	.11	1.00 (0.99-1.00)	.01	1.00 (0.99-1.01)	.99
Health spending per capita^c^	0.97 (0.93-1.00)	.07	0.97 (0.95-1.00)	.02	1.01 (0.95-1.06)	.85
Non-Hispanic Black population^d^	1.00 (0.99-1.00)	.70	1.00 (0.99-1.00)	.48	1.00 (1.00-1.00)	.86
High school graduation rate^e^	0.99 (0.99-1.00)	.006	1.00 (0.99-1.00)	.04	0.99 (0.99-1.00)	.001
Medicaid expansion	0.96 (0.92-0.99)	.009	0.97 (0.95-1.00)	.07	0.97 (0.92-1.02)	.21
Multivariable^f^						
Tax revenue per capita^a^	0.98 (0.95-0.98)	<.001	0.97 (0.95-0.98)	<.001	0.99 (0.98-1.02)	.87

Abbreviation: aIRR, adjusted incidence rate ratio.

^a^ Year dummy variables are not displayed.

^b^ American Indian or Alaska Native, Asian or Pacific Islander, and Black or African American (1128 state-years).

^c^ For all revenue and spending variables, the aIRR applies to a $1000 increase in revenue per capita.

^d^ aIRR applies to a 10% increase in percentage population.

^e^ aIRR applies to a 5% increase in graduation rate.

^f^ Adjusted for federal transfer revenue per capita, other revenue per capita, non-Hispanic Black population percentage, high school graduation rate, health spending per capita, and Medicaid expansion (variance inflation factor <5).

Specifically, among cancers with guideline-recommended screening, each $1000 increase in tax revenue per capita was associated with a 4% decrease in mortality rate (aIRR, 0.96; 95% CI, 0.94-0.98). Secondary analyses found that among cancers with established screening programs, each $1000 increase in tax-revenue per capita was associated with a 5% decrease in mortality among White populations (aIRR, 0.95; 95% CI, 0.93-0.98). In contrast, there was no association of tax revenue per capita with cancer mortality among racial and ethnic minority populations (aIRR, 0.99; 95% CI, 0.97-1.02) (Table 4). For some state-years, cancer mortality data for racial and ethnic minority populations were missing because deaths counts were suppressed for state-years reporting fewer than 16 deaths.

**Table 4.** Models for Cancer Mortality Rates (1999-2021) Among Cancers With Guideline-Recommended Screening (Colorectal, Breast, and Cervical) With a 2-Year Lag (1150 State-Years)^a^

Model	Cancer mortality					
	All, aIRR (95% CI)	*P* value	White, aIRR (95% CI)	*P* value	Racial and ethnic minority, aIRR (95% CI)^b^	*P* value
Univariable						
Tax revenue per capita^c^	0.90 (0.87-0.94)	<.001	0.90 (0.87-0.94)	<.001	0.98 (0.88-1.10)	.54
Federal transfer revenue per capita^c^	0.85 (0.81-0.89)	<.001	0.85 (0.81-0.89)	<.001	0.94 (0.90-0.98)	.008
Other revenue per capita^c^	0.99 (0.99-0.99)	<.001	0.98 (0.97-0.99)	<.001	0.91 (0.78-1.07)	.28
Health spending per capita^c^	0.89 (0.81-0.97)	.006	0.89 (0.82-0.96)	.002	0.97 (0.87-1.07)	.49
Non-Hispanic Black population^d^	0.99 (0.99-0.99)	.001	1.00 (1.00-1.00)	.16	0.99 (0.99-1.00)	.001
High school graduation rate^e^	0.97 (0.97-0.98)	.001	0.97 (0.96-0.98)	<.001	0.99 (0.98-0.99)	.001
Medicaid expansion	0.94 (0.90-0.99)	.02	0.95 (0.90-1.00)	.03	0.99 (0.95-1.03)	.53
Multivariable^f^						
Tax revenue per capita^c^	0.96 (0.94-0.98)	.001	0.95 (0.93-0.98)	<.001	0.99 (0.97-1.02)	.50

Abbreviation: aIRR, adjusted incidence rate ratio.

^a^ Generalized estimating equations models using Poisson distribution (log link) with robust standard errors and an exchangeable correlation matrix. Year dummy variables are not displayed.

^b^ American Indian or Alaska Native, Asian or Pacific Islander, and Black or African American (988 state-years).

^c^ For all revenue and spending variables, the aIRR applies to a $1000 increase in revenue per capita.

^d^ aIRR applies to a 10% increase in percentage population.

^e^ aIRR applies to a 5% increase in graduation rate.

^f^ Adjusted for federal transfer revenue per capita, other revenue per capita, non-Hispanic Black population percentage, high school graduation rate, health spending per capita, and Medicaid expansion (variance inflation factor <5).

## Discussion

To date, only a few studies have evaluated associations of tax policy with mortality in the US, with most reports focusing on infant and maternal mortality.^15,40,41,42,43,44^ Therefore, this cross-sectional study was important because we examined the association of state-level tax revenue per capita and the Suits index of tax progressivity with screening and cancer mortality rates throughout the US. Of note, there was a marked variation in the total revenue that state governments collected from taxes, fees, and charges, which reflected the diversity and federalist traditions in the US. Similarly, state-level spending on public goods and services, including health services, varied significantly.^36^ Although redistribution of wealth and privilege at the state level can be mediated by both spending and taxation, existing literature has predominantly evaluated the role of spending.^31,45^ The current study specifically evaluated tax policy and found that increased state-level tax revenue was associated with both increased colon, breast, and cervical cancer screening, as well as decreased cancer mortality. Of note, the observed associations varied among different racial and ethnic populations.

The conceptual framework linking tax policy to improved health outcomes is based on the potential of tax systems to enhance representation and promote democratic accountability.^46^ Tax payers contribute to the creation of public revenue, thus becoming responsible for holding governments accountable and demanding high quality goods and services, including health services.^47^ Moreover, state governments use tax revenue to finance equitable health care, helping to address some of the inherent inequalities of a privately financed health care system.^46^ Additionally, the revenue stream generated by taxation can fund public health care, as well as other public services that promote health-related goals. In fact, Barnes et al^45^ found that state-level public assistance was associated with improved survival among patients with cancer. Overall, investments in public services, infrastructure, environmental conditions, systems of transportation, education, and parks and recreation have been shown to improve health outcomes among the entire community, while being particularly beneficial to racial and ethnic minority populations and low-income individuals.^44,48^

Tax policy as an SDoH has been increasingly regarded as a vital, valid public health measure, allowing for the promotion of fairness and wealth redistribution within society.^46^ The association of tax policy with mortality was recently demonstrated by Junior et al,^15^ who reported that higher revenue and more progressive taxes were associated with decreased infant mortality rates. In the current study, state-level tax revenue per capita was associated with cancer screening and mortality rates. Specifically, incremental increases in tax revenue per capita were associated with corresponding increases in cancer screening rates and declines in the incidence of cancer mortality. These findings are in line with previous studies^43,49^ reporting that increased tax revenue and higher progressivity may lead to improved welfare and health care outcomes. Tax revenue may serve as funding that promotes the common good by ensuring access to safe, healthy environments and quality health care, while progressive taxes can substantially increase the disposable income of working-class households, thus enhancing their living standards and improving their health and cancer outcomes.^40,42^

Of note, inequities may persist even when there is an overall observed improvement toward a health goal, in which the improvement is not equitably distributed throughout the community.^8^ This finding was evident in the current study, in which increased tax revenue was associated with lower cancer mortality among White populations, yet this association was absent for racial and ethnic minority populations. Similarly, for cancers with guideline-recommended screening, higher tax revenue per capita was associated with cancer mortality in the overall study cohort and among White individuals; however, no such association was observed among racially and ethnically minoritized populations. Importantly, Black and Hispanic populations have historically faced discrimination and limited access to social resources, thus being exposed to worse environmental conditions, socioeconomic challenges, and limited educational and employment opportunities.^9^ SDoH represent obstacles to quality health care, with various studies demonstrating the impact of these factors on cancer prevention strategies.^2,9,45,50^ Furthermore, low income, low health literacy, and long distances to screening and treatment sites, as well as lack of health insurance, transportation means, or paid medical leave have been associated with decreased likelihood of undergoing timely screenings or receiving guideline-appropriate cancer care.^8,50^ To this end, findings of the current study highlight that although higher tax revenue may improve cancer-related outcomes, the benefit does not appear to affect racial and ethnic minority and disadvantaged populations. Importantly, because 97.6% of state-years in the current study had regressive tax policies, even high tax revenue may disproportionately burden racially and ethnically minoritized communities who are required to devote large percentages of their income to state and local taxes. Notably, the most regressive policies in the US were in Southern states, which have historically had higher concentrations of racial and ethnic minority populations compared with other regions (Figure, B).^51^ As such, while greater tax revenue may fund essential investments in housing, education, income, and transportation, the very populations that stand to benefit the most are also taxed at relatively higher rates, thus limiting their capacity to access these resources.^9,17,44,52^

Obstacles to accessing health care can lead to late diagnosis and worse prognosis, with Black populations demonstrating the highest overall cancer death rates. Black women present higher likelihood of death from breast cancer compared with White women, in addition to the highest cervical cancer death rates overall.^8^ Furthermore, screening rates are usually lower among racial and ethnic minority populations, uninsured individuals, and residents of rural areas.^53^ To this end, various cancer screening evidence-based programs have been designed and executed in recent years, partially funded by state governments, focusing on increasing screening rates specifically among racial and ethnic minority and low-income populations.^53^ For instance, the Community Cancer Screening Program aimed to increase colorectal cancer screening rates among low income, underinsured, or uninsured individuals in medically underserved, rural communities in Georgia, while the goal of the New Hampshire Colorectal Cancer Screening Program was to implement quality screening for low-income, uninsured, and underinsured patients.^53,54,55^ Of note, by prioritizing outreach to racial and ethnic minority and uninsured populations, the Delaware Cancer Consortium nearly eliminated disparities in screening, incidence, and advanced staged diagnosis of colorectal cancer among African American individuals within a 7-year period.^56^ Similarly, the Citywide Colon Cancer Control Coalition in New York initiated a colonoscopy screening campaign in 2003, reporting decreasing trends in both incidence and mortality among all racial and ethnic subgroups over the subsequent 13 years.^57^ In North Carolina, the North Carolina Breast Cancer Screening Program promoted awareness and ensured compliance among African American women, while the Forsyth County Cancer Screening Project increased breast and cervical cancer screening rates among low-income, Black women via the implementation of both clinic in-reach and community outreach strategies.^58,59^ In addition, Kukui Ahi (Light the Way) in Hawaii and the Targeting Cancer in Blacks program in Tennessee and Georgia successfully boosted overall screening rates for colorectal, cervical, breast and prostate cancer among Asian and Pacific Islander and Black adults, respectively.^60,61^ These evidence-based screening programs represent successful state-level initiatives and should highlight how government allocation of revenue can advance health care and cancer prevention goals.

### Limitations

There are several limitations to consider when interpreting the results of the current study. The ecological profile of the study meant that we could not control for potential individual-level confounders, nor draw causal inferences. Furthermore, there may be potential measurement errors in the external, publicly available data used. Tax progressivity was not included in all years evaluated in the current study due to only 5 years of ITEP tax progressivity data being reported within the defined study period. The study was conducted at the state level due to tax policy data being available at that geographic level, thus potentially masking more granular variations. Future studies should aim to examine the association of taxation with cancer-related outcomes at more granular levels. The analysis did not account for potential nonlinear threshold effects, namely effects of tax policy on cancer screening or cancer mortality that occurred solely over or under a level of revenue or progressivity. Although the use of 2- and 4-year lags between tax policy and outcomes is based on existing literature, the actual time required for tax policy to impact health- and cancer-related outcomes remains unclear.^15,41,62^ Cancer screening rates were estimated based on data from self-reported questionnaires, potentially introducing bias to the reported estimates.

## Conclusions

In conclusion, tax policy represents an SDoH that may impact cancer screening and cancer mortality rates in the US, based on the height of the tax revenue and the progressivity of the taxes. Therefore, designing tax systems based on high tax revenue and progressive policies may present one aspect of a multifactorial approach to improve cancer-related outcomes, thus mitigating persistent cancer health care inequalities in the US.

## Notes

Supplement 1. **eFigure 1.** Flowchart Demonstrating the Construction of the Unit of the Analysis (State-Year) and the Respective Sample Size for Each Outcome **eMethods.** Description of Screening Data, Covariate Definition, Selection of Generalized Estimating Equations Models and Covariate Exclusion **eTable 1.** Quasi-Likelihood Under the Independence Model Criterion (QIC) Values for different Generalized Estimating Equation (GEE) Model Distribution and Correlation Structures **eFigure 2.** Histograms Demonstrating the Distribution of Primary Outcomes of State-Level Colorectal Cancer Screening, Breast Cancer Screening, Cervical Cancer Screening and Death Counts due to Cancer **eFigure 3.** Pearson Correlation Matrix Among All Covariates Included in the Analysis **eTable 2.** Sensitivity Analysis: Univariable and Multivariable Generalized Estimating Equations Models for Cancer Mortality Rates (1999-2021) with 3-, 4-, and 5-Year Lag (1150 state-Years) Using Poisson Distribution (Log Link) With Robust Standard Errors and an Exchangeable Correlation Matrix **eTable 3.** Sensitivity Analysis: Multivariable Generalized Estimating Equations Models for Cancer Mortality Rates (1999-2021) Using Poisson Distribution (Log Link) With Robust Standard Errors and an Exchangeable Correlation Matrix and State-Fixed Effects **eTable 4.** Univariable and Multivariable Generalized Estimating Equations Models for Cancer Mortality Rates (2002, 2009, 2012, 2014, 2018) With 2-Year Lag (250 State-Years) Using Poisson Distribution (Log Link) With Robust Standard Errors and an Exchangeable Correlation Matrix
eReferences

Supplement 2.
Data Sharing Statement

## References

[1] Healthy People 2030. Reduce the overall cancer death rate—C-01. Office of Disease Prevention and Health Promotion. Accessed October 6, 2024. https://health.gov/healthypeople/objectives-and-data/browse-objectives/cancer/reduce-overall-cancer-death-rate-c-01

[2] Healthy People 2030. Cancer. Office of Disease Prevention and Health Promotion. Accessed October 6, 2024. https://health.gov/healthypeople/objectives-and-data/browse-objectives/cancer

[3] Healthy People 2030. Increase the proportion of females who get screened for breast cancer—C-05. Office of Disease Prevention and Health Promotion. Accessed October 6, 2024. https://health.gov/healthypeople/objectives-and-data/browse-objectives/cancer/increase-proportion-females-who-get-screened-breast-cancer-c-05

[4] Healthy People 2030. Increase the proportion of females who get screened for cervical cancer—C-09. Office of Disease Prevention and Health Promotion. Accessed October 6, 2024. https://health.gov/healthypeople/objectives-and-data/browse-objectives/cancer/increase-proportion-females-who-get-screened-cervical-cancer-c-09

[5] Healthy People 2030. Increase the proportion of adults who get screened for colorectal cancer—C-07. Office of Disease Prevention and Health Promotion. Accessed October 6, 2024. https://health.gov/healthypeople/objectives-and-data/browse-objectives/cancer/increase-proportion-adults-who-get-screened-colorectal-cancer-c-07

[6] Centers for Disease Control and Prevention. Leading cancer cases and deaths, all races and ethnicities, male and female, 2021. Published June 2024. Accessed October 7, 2024. https://gis.cdc.gov/Cancer/USCS/?CDC_AA_refVal=https%3A%2F%2Fwww.cdc.gov%2Fcancer%2Fdataviz%2Findex.htm#/AtAGlance/

[7] Ward EM, Sherman RL, Henley SJ, . Annual report to the nation on the status of cancer, featuring cancer in men and women age 20-49 years. J Natl Cancer Inst. 2019;111(12):1279-1297. doi:10.1093/jnci/djz106 31145458 PMC6910179

[8] National Cancer Institute. Cancer disparities. National Institutes of Health. Updated January 31, 2025. Accessed March 28, 2025. https://www.cancer.gov/about-cancer/understanding/disparities

[9] Munir MM, Woldesenbet S, Alaimo L, . Mediators of county-level racial and economic privilege in cancer screening. J Surg Oncol. 2023;127(7):1212-1222. doi:10.1002/jso.27238 36932957

[10] Munir MM, Woldesenbet S, Endo Y, . Racial segregation among patients with cholangiocarcinoma-impact on diagnosis, treatment, and outcomes. Ann Surg Oncol. 2023;30(7):4238-4246. doi:10.1245/s10434-023-13122-1 36695990

[11] Chatzipanagiotou OP, Woldesenbet S, Munir MM, . Impact of contemporary redlining on healthcare disparities among patients with gastrointestinal cancer: a mediation analysis. Ann Surg Oncol. 2025;32(2):1199-1209. doi:10.1245/s10434-024-16373-8 39485616 PMC11698888

[12] Chinaemelum A, Munir MM, Azap L, . Impact of Food insecurity on outcomes following resection of hepatopancreaticobiliary cancer. Ann Surg Oncol. 2023;30(9):5365-5373. doi:10.1245/s10434-023-13723-w 37314542

[13] Vutien P, Shah R, Ma K, Saleem N, Melson J. Utilization of census tract-based neighborhood poverty rates to predict non-adherence to screening colonoscopy. Dig Dis Sci. 2019;64(9):2505-2513. 30874988 10.1007/s10620-019-05585-8

[14] Moazzam Z, Woldesenbet S, Endo Y, . Association of historical redlining and present-day social vulnerability with cancer screening. J Am Coll Surg. 2023;237(3):454-464. doi:10.1097/XCS.0000000000000779 37318132

[15] Junior JA, Lee LK, Fleegler EW, Monuteaux MC, Niescierenko ML, Stewart AM. Association of state-level tax policy and infant mortality in the United States, 1996-2019. JAMA Netw Open. 2023;6(4):e239646. doi:10.1001/jamanetworkopen.2023.9646 37093600 PMC10126872

[16] Wiehe M, Davis A, Davis C, Gardner M, Gee L, Grundman D. Who Pays? A distributional analysis of the tax systems in all 50 states: sixth edition. Institute on Taxation and Economic Policy. Published October 2018. Accessed March 28, 2025. https://itep.sfo2.digitaloceanspaces.com/whopays-ITEP-2018.pdf

[17] Davis C, Byerly-Duke E, Ettlinger M, Who Pays? A distributional analysis of the tax systems in all 50 states: seventh edition. Institute on Taxation and Economic Policy. Published January 2024. Accessed March 28, 2025. https://sfo2.digitaloceanspaces.com/itep/ITEP-Who-Pays-7th-edition.pdf

[18] Arno PS, Sohler N, Viola D, Schechter C. Bringing health and social policy together: the case of the earned income tax credit. J Public Health Policy. 2009;30(2):198-207. doi:10.1057/jphp.2009.3 19597453 PMC3148586

[19] Wright A, Smith KE, Hellowell M. Policy lessons from health taxes: a systematic review of empirical studies. BMC Public Health. 2017;17(1):583. doi:10.1186/s12889-017-4497-z 28629470 PMC5477308

[20] Vandenbroucke JP, von Elm E, Altman DG, ; STROBE Initiative. Strengthening the Reporting of Observational Studies in Epidemiology (STROBE): explanation and elaboration. PLoS Med. 2007;4(10):e297. doi:10.1371/journal.pmed.0040297 17941715 PMC2020496

[21] Davis C, Davis K, Gardner M, . Who Pays? A distributional analysis of the tax systems in all 50 states: fifth edition. Published January 2015. Accessed March 28, 2025. https://itep.sfo2.digitaloceanspaces.com/whopaysreport.pdf

[22] Davis C, Davis K, Gardner M, . Who Pays? A distributional analysis of the tax systems in all 50 states: fourth edition. Published January 2013. Accessed March 28, 2025. https://itep.sfo2.digitaloceanspaces.com/whopaysreport4th.pdf

[23] Davis C, Davis K, Gardner M, McIntyre RS, McLynch J, Sapozhnikova A. Who Pays? A distributional analysis of the tax systems in all 50 states: third edition. Published November 2009. Accessed March 28, 2025. https://itep.sfo2.digitaloceanspaces.com/whopays3.pdf

[24] McIntyre RS, Denk R, Francis N, Who Pays? A distributional analysis of the tax systems in all 50 states: second edition. Published January 2003. Accessed March 28, 2025. https://itep.sfo2.digitaloceanspaces.com/wp2003.pdf

[25] Ettlinger MP, McIntyre RS, Fray EA, O’ Hare JF, King J, Miransky N. Who Pays? A Distributional Analysis of the Tax Systems in All 50 States: first edition. Published June 1996. Accessed March 28, 2025. https://itep.sfo2.digitaloceanspaces.com/wp1996.pdf

[26] U.S. Census Bureau. Government finances data: data sets. Updated October 9, 2024. Accessed December 26, 2024. https://www.census.gov/programs-surveys/gov-finances/data/datasets.html

[27] Bureau of Economic Analysis. Data archive: national accounts. Accessed October 7, 2024. https://apps.bea.gov/histdatacore/

[28] Centers for Disease Control and Prevention. US cancer statistics: mortality data, 1999-2021. Updated March 26, 2025. Accessed March 28, 2025. https://wonder.cdc.gov/cancer.html

[29] Centers for Disease Control and Prevention. PLACES: local data for better health. Accessed September 12, 2024. https://www.cdc.gov/places/index.html

[30] US Census Bureau. Annual survey of state and local government finances. Updated February 5, 2025. Accessed March 28, 2025. https://www.census.gov/programs-surveys/gov-finances.html

[31] O’Brien RL. Redistribution and the new fiscal sociology: race and the progressivity of state and local taxes. AJS. 2017;122(4):1015-1049. doi:10.1086/690118 30135607 PMC6101670

[32] Piatkowska SJ, Messner SF, Gruner C, Baumer EP. The “new fiscal criminology”: state-level changes in crime rates and the structure of tax systems. Justice Q. 2022;39(2):304-326. doi:10.1080/07418825.2020.1731572

[33] Suits DB. Measurement of tax progressivity. *Am Econ Rev*. 1977;67(4):747-752

[34] Arcarons J, Calonge S. Inference tests for tax progressivity and income redistribution: the Suits approach. J Econ Inequal. Published online June 15, 2014. doi:10.1007/s10888-014-9280-0

[35] US Commission on Civil Rights. Targeted fines and fees against communities of color: civil rights & constitutional implications. Published 2017. Accessed March 28, 2025. https://www.usccr.gov/files/pubs/2017/Statutory_Enforcement_Report2017.pdf

[36] Gordon T, Auxier RC, Iselin J. Assessing fiscal capacities of states: a representative revenue system–representative expenditure system approach, fiscal year 2012. Published March 7, 2016. Accessed March 28, 2025. https://www.urban.org/research/publication/assessing-fiscal-capacities-states-representative-revenue-system-representative-expenditure-system-approach-fiscal-year-2012

[37] French B, Stuart EA. Study designs and statistical methods for studies of child and adolescent health policies. JAMA Pediatr. 2020;174(10):925-927. doi:10.1001/jamapediatrics.2020.3408 32897312 PMC7986989

[38] Ghisletta P, Spini D. An introduction to generalized estimating equations and an application to assess selectivity effects in a longitudinal study on very old individuals. J Educ Behav Stat. 2004;29(4):421-437. doi:10.3102/10769986029004421

[39] Python Core Team. Python 3.12: a dynamic, open source programming language. Python Software Foundation. Accessed March 28, 2025. https://www.python.org/

[40] MacKenzie TA, Houle J, Jiang S, Onega T. Middle-aged death and taxes in the USA: association of state tax burden and expenditures in 2005 with survival from 2006 to 2015. PLoS One. 2019;14(4):e0214463. doi:10.1371/journal.pone.0214463 30978199 PMC6461276

[41] Rigby E, Hatch ME. Incorporating economic policy into a ‘health-in-all-policies’ agenda. Health Aff (Millwood). 2016;35(11):2044-2052. doi:10.1377/hlthaff.2016.0710 27834245

[42] Kim AS, Jennings ET. Effects of U.S. states’ social welfare systems on population health. Policy Stud J. 2009;37(4):745-767. doi:10.1111/j.1541-0072.2009.00333.x

[43] Granruth LB, Shields JJ. Impact of the level of state tax code progressivity on children’s health outcomes. Health Soc Work. 2011;36(3):207-215. doi:10.1093/hsw/36.3.207 21936334

[44] Vilda D, Walker BC, Hardeman RR, Wallace ME. Associations between state and local government spending and pregnancy-related mortality in the U.S. Am J Prev Med. 2023;64(4):459-467. doi:10.1016/j.amepre.2022.10.022 36658021 PMC10033388

[45] Barnes JM, Johnston KJ, Johnson KJ, Chino F, Osazuwa-Peters N. State public assistance spending and survival among adults with cancer. JAMA Netw Open. 2023;6(9):e2332353. doi:10.1001/jamanetworkopen.2023.32353 37669050 PMC10481229

[46] Mccoy D, Chigudu S, Tillmann T. Framing the tax and health nexus: a neglected aspect of public health concern. Health Econ Policy Law. 2017;12(2):179-194. doi:10.1017/S174413311600044X 28332460

[47] Prichard W. Taxation and state building: towards a governance focused tax reform agenda. GSRDC. Published 2010. Accessed March 28, 2025. https://gsdrc.org/document-library/taxation-and-state-building-towards-a-governance-focused-tax-reform-agenda/

[48] Bradley EH, Canavan M, Rogan E, . Variation in health outcomes: the role of spending on social services, public health, and health care, 2000-09. Health Aff (Millwood). 2016;35(5):760-768. doi:10.1377/hlthaff.2015.0814 27140980

[49] Kim D. The associations between US state and local social spending, income inequality, and individual all-cause and cause-specific mortality: the national longitudinal mortality study. Prev Med. 2016;84:62-68. doi:10.1016/j.ypmed.2015.11.013 26607868 PMC5766344

[50] Bauer C, Zhang K, Xiao Q, Lu J, Hong YR, Suk R. County-level social vulnerability and breast, cervical, and colorectal cancer screening rates in the US, 2018. JAMA Netw Open. 2022;5(9):e2233429. doi:10.1001/jamanetworkopen.2022.33429 36166230 PMC9516325

[51] KFF (Kaiser Family Foundation). Population distribution by race/ethnicity. Published 2023. Accessed February 27, 2025. https://www.kff.org/other/state-indicator/distribution-by-raceethnicity/

[52] Bailey ZD, Krieger N, Agénor M, Graves J, Linos N, Bassett MT. Structural racism and health inequities in the USA: evidence and interventions. Lancet. 2017;389(10077):1453-1463. doi:10.1016/S0140-6736(17)30569-X 28402827

[53] Division of Cancer Control and Population Sciences. Community cancer screening program (CCSP). National Cancer Institute. Updated April 20, 2023. Accessed October 12, 2024. https://ebccp.cancercontrol.cancer.gov/programDetails.do?programId=24355707

[54] Division of Cancer Control and Population Sciences. Against Colorectal Cancer in Our Neighborhoods (ACCION). National Cancer Institute. Updated January 3, 2024. Accessed October 12, 2024. https://ebccp.cancercontrol.cancer.gov/programDetails.do?programId=26767808

[55] Division of Cancer Control and Population Sciences. New Hampshire colorectal cancer screening program (NHCRCSP) patient navigation intervention. National Cancer Institute. Updated April 25, 2024. Accessed October 12, 2024. https://ebccp.cancercontrol.cancer.gov/programDetails.do?programId=27435365

[56] Grubbs SS, Polite BN, Carney J Jr, . Eliminating racial disparities in colorectal cancer in the real world: it took a village. J Clin Oncol. 2013;31(16):1928-1930. doi:10.1200/JCO.2012.47.8412 23589553 PMC3661932

[57] Brown JJ, Asumeng CK, Greenwald D, . Decreased colorectal cancer incidence and mortality in a diverse urban population with increased colonoscopy screening. BMC Public Health. 2021;21(1):1280. doi:10.1186/s12889-021-11330-6 34193094 PMC8247120

[58] Division of Cancer Control and Population Sciences. The Forsyth County cancer screening project (FoCaS). National Cancer Institute. Updated July 21, 2020. Accessed October 13, 2024. https://ebccp.cancercontrol.cancer.gov/programDetails.do?programId=141840

[59] Division of Cancer Control and Population Sciences. North Carolina breast cancer screening program. National Cancer Institute. Updated March 21, 2024. Accessed October 13, 2024. https://ebccp.cancercontrol.cancer.gov/programDetails.do?programId=566594

[60] Division of Cancer Control and Population Sciences. Kukui Ahi (Light the Way): patient navigation. National Cancer Institute. Updated April 20, 2023. Accessed October 12, 2024. https://ebccp.cancercontrol.cancer.gov/programDetails.do?programId=26140843

[61] Division of Cancer Control and Population Sciences. Targeting cancer in Blacks (TCiB). National Cancer Institute. Updated December 5, 2023. Accessed October 13, 2024. https://ebccp.cancercontrol.cancer.gov/programDetails.do?programId=310347

[62] Goldstein ND, Palumbo AJ, Bellamy SL, Purtle J, Locke R. State and local government expenditures and infant mortality in the United States. Pediatrics. 2020;146(5):e20201134. doi:10.1542/peds.2020-1134 33077541

