# Supplementary material for: State-Level Tax Policy, Cancer Screening, and Mortality Rates in the US
Source: JAMA Netw Open. 2025 May 2;8(5):e258455. doi: 10.1001/jamanetworkopen.2025.8455 (PMC12048849; doi:10.1001/jamanetworkopen.2025.8455)
Supplement: Supplement 1. — eFigure 1. Flowchart Demonstrating the Construction of the Unit of the Analysis (State-Year) and the Respective Sample Size for Each Outcome eMethods. Description of Screening Data, Covariate Definition, Selection of Generalized Estimating Equations Models and Covariate Exclusion eTable 1. Quasi-Likelihood Under the Independence Model Criterion (QIC) Values for different Generalized Estimating Equation (GEE) Model Distribution and Correlation Structures eFigure 2. Histograms Demonstrating the Distribution of Primary Outcomes of State-Level Colorectal Cancer Screening, Breast Cancer Screening, Cervical Cancer Screening and Death Counts due to Cancer eFigure 3. Pearson Correlation Matrix Among All Covariates Included in the Analysis eTable 2. Sensitivity Analysis: Univariable and Multivariable Generalized Estimating Equations Models for Cancer Mortality Rates (1999-2021) with 3-, 4-, and 5-Year Lag (1150 state-Years) Using Poisson Distribution (Log Link) With Robust Standard Errors and an Exchangeable Correlation Matrix eTable 3. Sensitivity Analysis: Multivariable Generalized Estimating Equations Models for Cancer Mortality Rates (1999-2021) Using Poisson Distribution (Log Link) With Robust Standard Errors and an Exchangeable Correlation Matrix and State-Fixed Effects eTable 4. Univariable and Multivariable Generalized Estimating Equations Models for Cancer Mortality Rates (2002, 2009, 2012, 2014, 2018) With 2-Year Lag (250 State-Years) Using Poisson Distribution (Log Link) With Robust Standard Errors and an Exchangeable Correlation Matrix eReferences [file jamanetwopen-e258455-s001.pdf]

Supplemental Online Content

Chatzipanagiotou OP, Khalil M, Waqar U, Woldesenbet S, Catalano G, Pawlik TM. State-level tax policy, cancer screening, and mortality rates in the US. *JAMA Netw. Open*. 2025;8(5):e258455. doi:10.1001/jamanetworkopen.2025.8455

eFigure 1. Flowchart Demonstrating the Construction of the Unit of the Analysis (State-Year) and the Respective Sample Size for Each Outcome

eMethods. Description of Screening Data, Covariate Definition, Selection of Generalized Estimating Equations Models and Covariate Exclusion

eTable 1. Quasi-Likelihood Under the Independence Model Criterion (QIC) Values for different Generalized Estimating Equation (GEE) Model Distribution and Correlation Structures

eFigure 2. Histograms Demonstrating the Distribution of Primary Outcomes of State-Level Colorectal Cancer Screening, Breast Cancer Screening, Cervical Cancer Screening and Death Counts due to Cancer

eFigure 3. Pearson Correlation Matrix Among All Covariates Included in the Analysis

eTable 2. Sensitivity Analysis: Univariable and Multivariable Generalized Estimating Equations Models for Cancer Mortality Rates (1999-2021) with 3-, 4-, and 5-Year Lag (1150 state-Years) Using Poisson Distribution (Log Link) With Robust Standard Errors and an Exchangeable Correlation Matrix

eTable 3. Sensitivity analysis: Multivariable Generalized Estimating Equations Models for Cancer Mortality Rates (1999-2021) Using Poisson Distribution (Log Link) With Robust Standard Errors and an Exchangeable Correlation Matrix and State-Fixed Effects

eTable 4. Univariable and multivariable Generalized Estimating Equations Models for Cancer Mortality Rates (2002, 2009, 2012, 2014, 2018) With 2-Year Lag (250 State-Years) Using Poisson Distribution (Log Link) With Robust Standard Errors and an Exchangeable Correlation Matrix

eReferences

This supplemental material has been provided by the authors to give readers additional information about their work.

© 2025 Chatzipanagiotou OP et al. *JAMA Network Open*.

eFigure 1. Flowchart demonstrating the construction of the unit of the analysis (state-year) and the respective sample size for each outcome

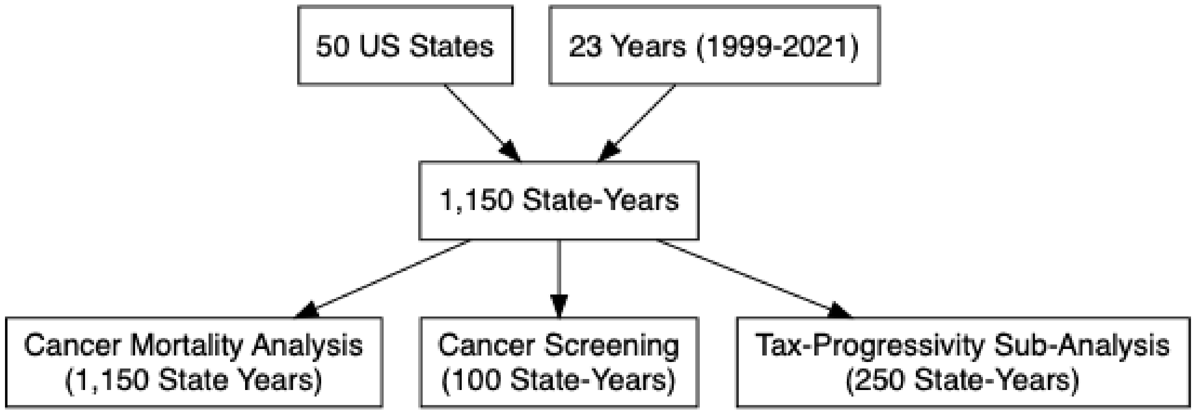

© 2025 Chatzipanagiotou OP et al. *JAMA Network Open*.

eMethods

Description of Screening Data

Colorectal, breast, and cervical cancer screening data were queried from the CDC Population Level Analysis and Community Estimates (PLACES) database. The calculation of these estimates is based on a combination of multi-level regression and post-stratification techniques for small area estimation. The 2020 PLACES data release uses data from the 2018 Behavioral Risk Factor Surveillance System (BRFSS), 2018 US Census Bureau, and 2014-2018 American Community Survey (ACS). The 2022 PLACES data release uses data from the 2020 BRFSS, 2020 US Census Bureau, and 2016-2020 ACS.[1,2]

Breast cancer screening probability represented the age-adjusted prevalence of females aged 50-74 years who had a mammogram within the 2 previous years.[3] Similarly, cervical screening probability was based on data for women 21-65 without a hysterectomy, either aged 21-29 who had a Papanicolaou (Pap) test within 3 years, or aged 30-65 who had a Pap test alone within 3 years and a human papilloma virus test alone or a co-test within 5 years.[3] The probability of colorectal cancer screening utilized data among adults 40-75 who had either a fecal occult blood test (1-year), or a FIT-DNA test (3-year), or a sigmoidoscopy (5-year), or a sigmoidoscopy (10-year) with a FIT test (1-year), or a colonoscopy (10-year), or a virtual colonoscopy (5-year).[3] Screening rate probabilities were applied to specific geographical population estimates to calculate screening prevalence.[1]

Covariate Definition

The federal transfer revenue is defined as the grants transferred from the federal government to enhance state and local budgets, primarily aiming to cover deficiencies between state generated tax revenues and expenditures.[4,5] The term “Other revenue” was used to encompass revenue from fees, fines, and government investments; thus any revenue other than state- or local-level tax revenue or federal funding.[4,6] Health spending was defined as the aggregate state-level spending towards public welfare, hospitals, and health.[7]

Selection of Generalized Estimating Equations Models and Covariate Exclusion

Gamma regression with a log link was employed for cancer screening, while a Poisson regression with robust standard errors (SE) and log link was used for cancer mortality. An exchangeable correlation structure proved [illegible] autoregressive structure, and was used rather than unstructured matrix in cases where GEE models did not converge.[5,8] We hypothesized that a 2-year lag would allow sufficient time for investments in healthcare, hospital infrastructure, and public welfare to be implemented, thereby enhancing resources for patients with cancer both in-hospital and in the community. The ICC relative to cancer mortality suggested that the year-to-year variation of the independent variables in the model was minimal.

A relatively high correlation (r=0.76) was observed between tax revenue per capita and GDP per capita (**eFigure 2**). Moreover, the VIF for GDP per capita exceeded the VIF threshold in the models, leading to its exclusion from multivariable analyses due to concerns about multicollinearity. [5]

Although, an unconditional fixed-effects model could control for time-invariant, unmeasured state-level confounders, this would introduce the incidental parameters issue.[5,9-11] Additionally, incorporating state fixed effects risks overparameterizing models with smaller sample sizes, such as the cancer screening models or sub-analyses limited to years with tax progressivity data. Specifically, this would require the inclusion of 49 dummy variables, alongside independent variables, covariates, and year dummy variables, resulting in a much lower observation-to-predictor ratio than the 10:1 ratio commonly recommended in the literature.[12] While conditional fixed-effects models have been proposed as better alternatives for studies with over 20 clusters, they fail to account for all predictors and are thus not considered true fixed-effects models.[5] In light of these limitations, generalized estimating equation (GEE) models remain a widely accepted approach for analyzing clustered data, as they account for within-cluster correlation without overparameterizing the model.[13]

© 2025 Chatzipanagiotou OP et al. *JAMA Network Open*.

eTable 1. Quasi-likelihood under the independence model criterion (QIC) values for different generalized estimating equation (GEE) model distribution and correlation structures

**GEE model**	**Colorectal Screening**	**Breast Screening**	**Cervical Screening**	**GEE model**	**Cancer Mortality**
	QIC	QIC	QIC		QIC
Gaussian (identity link)				Gamma (log link)	
Autoregressive	2573.45	1397.51	394.56	Exchangeable	46,030,008,791
Exchangeable	2573.36	1400.96	395.38	Unstructured	Did not converge
Gamma (log link)				Poisson (log link)	
Autoregressive	217.09	216.07	214.84	Exchangeable	6,687,458
Exchangeable	217.04	218.63	215.60	Unstructured	Did not converge
Gamma (inverse link)				Negative Binomial (log link(	
Autoregressive	217.98	216.06	214.83	Exchangeable	46,036,056,351
Exchangeable	217.05	218.63	215.59	Unstructured	Did not converge

© 2025 Chatzipanagiotou OP et al. *JAMA Network Open*.

eFigure 2. Histograms demonstrating the distribution of primary outcomes of state-level colorectal cancer screening, breast cancer screening, cervical cancer screening and death counts due to cancer.

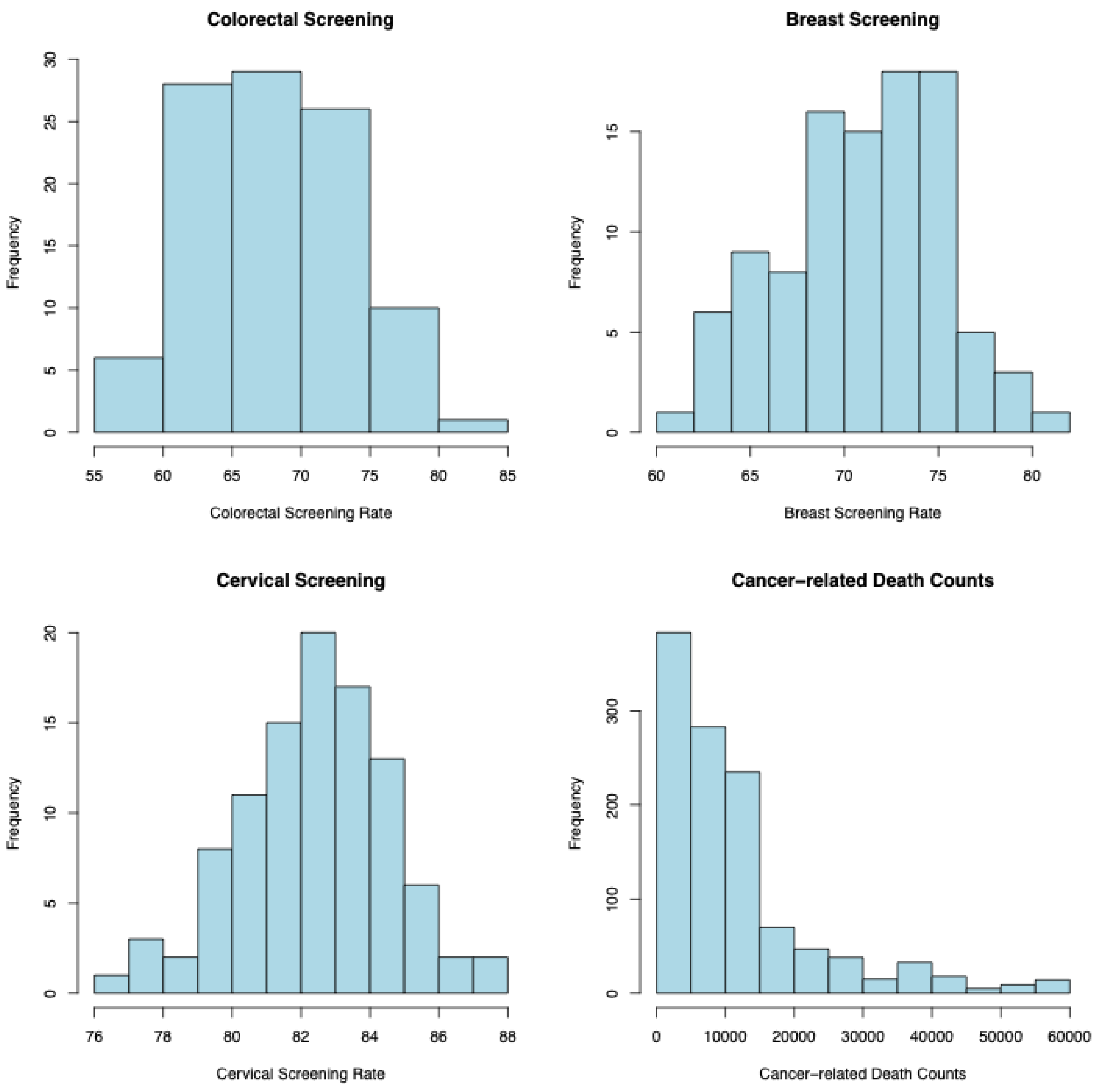

© 2025 Chatzipanagiotou OP et al. *JAMA Network Open*.

eFigure 3. Pearson correlation matrix among all covariates included in the analysis

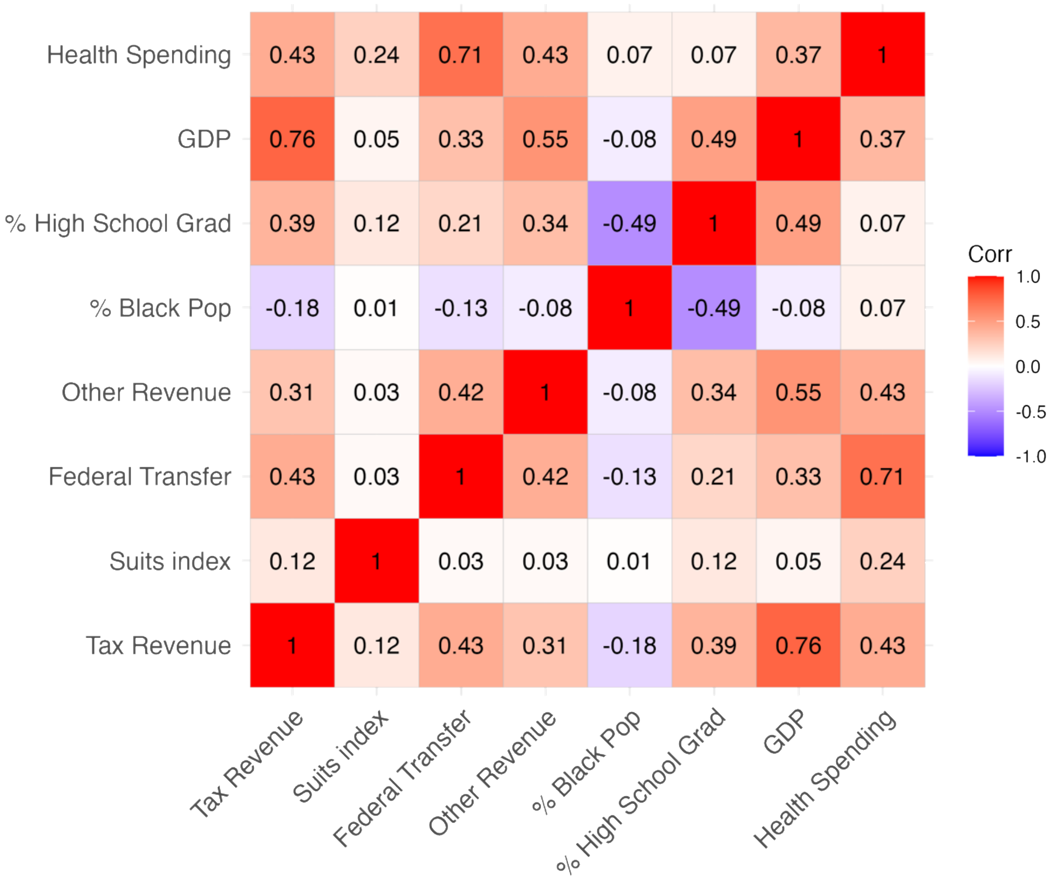

© 2025 Chatzipanagiotou OP et al. *JAMA Network Open*.

eTable 2. Sensitivity analysis - Univariable and multivariable generalized estimating equations models for cancer mortality rates (1999-2021) with 3-, 4-, and 5-year lag (1150 state-years) using Poisson distribution (log link) with robust standard errors and an exchangeable correlation matrix

All Cancer mortality, aIRRs (95%CI)

	3-year lag	p-value	4-year lag	p-value	5-year lag[f]	p-value
Univariable						
[a]Tax revenue per capita	0.96 (0.95 – 0.98)	<0.001	0.96 (0.95 – 0.98)	<0.001	0.97 (0.95 – 0.98)	<0.001
[a]Federal transfer revenue per capita	0.96 (0.93 – 0.98)	0.001	0.96 (0.94 – 0.99)	0.003	0.97 (0.94 – 0.99)	0.004
[a]Other revenue per capita	1.00 (0.99 – 1.00)	0.07	1.00 (0.99 – 1.00)	0.098	1.00 (0.99 – 1.00)	0.05
[a]Health spending per capita	0.97 (0.93 – 1.00)	0.05	0.97 (0.94 – 1.00)	0.055	0.97 (0.94 – 1.00)	0.05
[c]Non-Hispanic Black Population	0.99 (0.97 – 1.02)	0.54	0.99 (0.97 – 1.02)	0.49	0.99 (0.97 – 1.01)	0.46
[e]High School Graduation rate	0.99 (0.99 – 1.00)	0.004	0.99 (0.99 – 1.00)	0.003	0.99 (0.99 – 1.00)	0.002
Medicaid Expansion	0.96 (0.92 – 0.99)	0.009	0.96 (0.93 – 0.99)	0.009	0.96 (0.92 – 0.99)	0.009
[e]Multivariable						
[a]Tax revenue per capita	0.97 (0.95 – 0.98)	0.008	0.97 (0.95 – 0.98)	<0.001	0.97 (0.95 – 0.98)	<0.001

Abbreviations: aIIR, adjusted Incidence Rate Ratio; CI, Confidence Interval
[a]For all revenue and spending variables, the aIRR applies to $1000 increase in revenue per capita
[b]aIRR applies to +0.10 unit increase
[c]aIRR applies to a 10% increase in percentage population
[d]aIRR applies to a 5% increase in graduation rate
[e]Adjusted for Federal Tranfer Revenue per capita, Other Revenue per capita, Non-Hispanic Black Population %, High School Graduation Rate, Health Spending per capita, Medicaid Expansion (Variance Inflation Factor < 5)
[f]1100 state years for 5-year lag
Year dummy variables are not displayed

© 2025 Chatzipanagiotou OP et al. *JAMA Network Open*.

eTable 3. Sensitivity analysis - Multivariable generalized estimating equations models for cancer mortality rates (1999-2021) using Poisson distribution (log link) with robust standard errors and an exchangeable correlation matrix and state-fixed effects

	All Cancer mortality, aIRRs (95%CI)			
	All years	p-value	ITEP years	p-value
[c]Multivariable				
[a]Tax revenue per capita	0.97 (0.95 – 0.98)	<0.001	0.97 (0.95 – 0.99)	0.001
[b]Tax progressivity	-		1.03 (1.00 – 1.06)	0.020

Abbreviations: aIIR, adjusted Incidence Rate Ratio; CI, Confidence Interval; ITEP, Institute on Taxation and Economic Policy
[a]For all revenue and spending variables, the aIRR applies to $1000 increase in revenue per capita
[b]aIRR applies to +0.10 unit increase
[c]Adjusted for Federal Tranfer Revenue per capita, Other Revenue per capita, Non-Hispanic Black Population %, High School Graduation Rate, Health Spending per capita, Medicaid Expansion (Variance Inflation Factor < 5)
Year and state dummy variables are not displayed

© 2025 Chatzipanagiotou OP et al. *JAMA Network Open*.

eTable 4. Univariable and multivariable generalized estimating equations models for cancer mortality rates (2002, 2009, 2012, 2014, 2018) with 2-year lag (250 state-years) using Poisson distribution (log link) with robust standard errors and an exchangeable correlation matrix

	Cancer mortality, aIRRs (95%CI)					
	All mortality	p-value	White mortality	p-value	Minority mortality	p-value
Univariable						
[a]Tax revenue per capita	0.98 (0.96 – 0.99)	<0.001	0.98 (0.97 – 0.99)	0.001	1.00 (0.98 – 1.01)	0.53
[b]Tax progressivity	0.98 (0.95 – 1.01)	0.16	0.99 (0.96 – 1.02)	0.38	1.01 (0.97 – 1.05)	0.49
[a]Federal transfer revenue per capita	0.97 (0.94 – 1.00)	0.02	0.98 (0.95 – 1.01)	0.15	0.99 (0.95 – 1.02)	0.49
[a]Other revenue per capita	1.00 (0.99 – 1.00)	0.03	1.00 (1.00 – 1.00)	0.21	1.00 (0.99 – 1.01)	0.59
[a]Health spending per capita	0.97 (0.94 – 1.01)	0.06	0.98 (0.96 – 1.00)	0.11	0.99 (0.96 – 1.03)	0.73
[c]Non-Hispanic Black Population	1.00 (0.99 – 1.00)	0.91	1.00 (1.00 – 1.01)	0.33	1.00 (1.00 – 1.01)	0.01
[e]High School Graduation rate	1.00 (0.99 – 1.00)	0.03	1.00 (0.99 – 1.00)	0.40	1.00 (0.99 – 1.00)	0.22
Medicaid Expansion	0.99 (0.97 – 1.02)	0.50	0.99 (0.96 – 1.02)	0.59	0.99 (0.97 – 1.01)	0.47
[e]Multivariable						
[a]Tax revenue per capita	0.97 (0.96 – 0.99)	<0.001	0.97 (0.96 – 0.99)	<0.001	0.99 (0.98 – 1.01)	0.36
[b]Tax progressivity	1.02 (1.00 – 1.04)	0.10	1.02 (1.00 – 1.04)	0.07	1.04 (1.00 – 1.08)	0.06

Abbreviations: aIIR, adjusted Incidence Rate Ratio; CI, Confidence Interval
[a]For all revenue and spending variables, the aIRR applies to $1000 increase in revenue per capita
[b]aIRR applies to +0.10 unit increase
[c]aIRR applies to a 10% increase in percentage population
[d]aIRR applies to a 5% increase in graduation rate
[e]Adjusted for Federal Tranfer Revenue per capita, Other Revenue per capita, Non-Hispanic Black Population %, High School Graduation Rate, Health Spending per capita, Medicaid Expansion (Variance Inflation Factor < 5)
Year dummy variables are not displayed
246 state-years for minorities

© 2025 Chatzipanagiotou OP et al. *JAMA Network Open*.

eReferences

1. Centers for Disease Control and Prevention. PLACES: Local Data for Better Health. Accessed September 12, 2024,
2. Munir MM, Woldesenbet S, Alaimo L, et al. Mediators of county-level racial and economic privilege in cancer screening. *J Surg Oncol*. 2023;127(7):1212-1222. doi:10.1002/jso.27238
3. Centers for Disease Control and Prevention. Prevention Measures Definitions. Accessed September 12, 2024,
4. Gordon T, Auxier RC, Iselin J. *Assessing Fiscal Capacities of States: A Representative Revenue System–Representative Expenditure System Approach, Fiscal Year 2012*. 2016. https://www.urban.org/research/publication/assessing-fiscal-capacities-states-representative-revenue-system-representative-expenditure-system-approach-fiscal-year-2012
5. Junior JA, Lee LK, Fleegler EW, Monuteaux MC, Niescierenko ML, Stewart AM. Association of State-Level Tax Policy and Infant Mortality in the United States, 1996-2019. *JAMA Network Open*. 2023;6(4):e239646. doi:10.1001/jamanetworkopen.2023.9646
6. Lhamon CE, Heriot G, Kirsanow PN, et al. *Targeted Fines and Fees Against Communities of Color: Civil Rights & Constitutional Implications*. 2017.
7. US Census Bureau. Annual survey of state and local government finances. Accessed October 6, 2024, https://www.census.gov/programs-surveys/gov-finances/data/datasets.All.html
8. Ghisletta P, Spini D. An Introduction to Generalized Estimating Equations and an Application to Assess Selectivity Effects in a Longitudinal Study on Very Old Individuals. *Journal of Educational and Behavioral Statistics*. 2004;29(4):421-437. doi:10.3102/10769986029004421
9. Fitzmaurice GM, Laird NM, Ware JH. Applied Longitudinal Analysis. *Wiley Series in Probability and Statistics*. 2011;doi:10.1002/9781119513469
10. Schempf AH, Kaufman JS. Accounting for context in studies of health inequalities: a review and comparison of analytic approaches. *Ann Epidemiol*. 2012;22(10):683-690. doi:10.1016/j.annepidem.2012.06.105
11. Hubbard AE, Ahern J, Fleischer NL, et al. To GEE or Not to GEE. *Epidemiology*. 2010;21(4):467-474. doi:10.1097/ede.0b013e3181caeb90
12. Hilbe JM. Modeling Count Data. 2014;doi:10.1017/cbo9781139236065
13. French B, Stuart EA. Study Designs and Statistical Methods for Studies of Child and Adolescent Health Policies. *JAMA Pediatrics*. 2020;174(10):925. doi:10.1001/jamapediatrics.2020.3408

© 2025 Chatzipanagiotou OP et al. *JAMA Network Open*.
