# Supplementary material for: State-Level Tax Policy, Cancer Screening, and Mortality Rates in the US
Source: JAMA Netw Open. 2025 May 2;8(5):e258455. doi: 10.1001/jamanetworkopen.2025.8455 (PMC12048849; doi:10.1001/jamanetworkopen.2025.8455)
Supplement: Supplement 2. — Data Sharing Statement [file jamanetwopen-e258455-s002.pdf]

Data Sharing Statement

Chatzipanagiotou. State-Level Tax Policy, Cancer Screening, and Mortality Rates in the US. *JAMA Netw Open*. Published May 02, 2025. doi:10.1001/jamanetworkopen.2025.8455

Data
Data available: No

Additional Information
Explanation for why data not available: State-level Tax data were provided by Dr Jean A. Junior, MD, MPhil upon request, and were enhanced with publicly available data from the US Census Bureau and the Bureau of Economic Analysis. Publicly available cancer mortality data were derived from the Centers for Disease Control and Prevention WONDER database. Publicly available cancer screening rates were derived from the CDC Population Level Analysis and Community Estimates database.
